# Multi-Platform Sequencing Approach Reveals a Novel Transcriptome Profile in Pseudorabies Virus

**DOI:** 10.3389/fmicb.2017.02708

**Published:** 2018-01-22

**Authors:** Norbert Moldován, Dóra Tombácz, Attila Szűcs, Zsolt Csabai, Michael Snyder, Zsolt Boldogkői

**Affiliations:** ^1^Department of Medical Biology, Faculty of Medicine, University of Szeged, Szeged, Hungary; ^2^Department of Genetics, School of Medicine, Stanford University, Stanford, CA, United States

**Keywords:** herpesvirus, pseudorabies virus, long-read sequencing, short-read sequencing, transcriptome analysis, RNA-sequencing

## Abstract

Third-generation sequencing is an emerging technology that is capable of solving several problems that earlier approaches were not able to, including the identification of transcripts isoforms and overlapping transcripts. In this study, we used long-read sequencing for the analysis of pseudorabies virus (PRV) transcriptome, including Oxford Nanopore Technologies MinION, PacBio RS-II, and Illumina HiScanSQ platforms. We also used data from our previous short-read and long-read sequencing studies for the comparison of the results and in order to confirm the obtained data. Our investigations identified 19 formerly unknown putative protein-coding genes, all of which are 5′ truncated forms of earlier annotated longer PRV genes. Additionally, we detected 19 non-coding RNAs, including 5′ and 3′ truncated transcripts without in-frame ORFs, antisense RNAs, as well as RNA molecules encoded by those parts of the viral genome where no transcription had been detected before. This study has also led to the identification of three complex transcripts and 50 distinct length isoforms, including transcription start and end variants. We also detected 121 novel transcript overlaps, and two transcripts that overlap the replication origins of PRV. Furthermore, *in silico* analysis revealed 145 upstream ORFs, many of which are located on the longer 5′ isoforms of the transcripts.

## Introduction

Pseudorabies virus (PRV) is an animal herpesvirus belonging to the subfamily of *Alphaherpesvirinae*. It causes significant economic losses; therefore, programs toward eradication have been launched throughout the world. PRV is a popular model organism that is applied in various fields of biology, such as studying the molecular pathogenesis of herpesviruses (Pomeranz et al., 2005), mapping the neural circuits by using PRV as a polysynaptic tract-tracing tool (Boldogkői et al., 2004; Nakamura et al., 2004; Ekstrand et al., 2008; Card and Enquist, 2014; Pomeranz et al., 2017), and delivering fluorescent activity markers to the brain through synapses (Boldogkői et al., 2009). PRV is also used as a model for the investigation of the transcription interference networks (Boldogkői, 2012).

Pseudorabies virus has a double-stranded DNA molecule with a size of approximately 142 kilobase pairs (Tombácz et al., 2014) containing 67 protein-coding and several non-coding transcripts (Tombácz et al., 2009). The viral genome harbors a unique long (UL) and a unique short (US) region, the latter being located within the two inverted repeats (IRs). Most of the PRV genes are expressed as polycistronic transcripts, which are typical in prokaryotes but very rare in eukaryotic organisms (Mainguy et al., 2007). Basically, PRV uses the host’s transcriptional apparatus for its RNA synthesis, but it also produces virus-specific transcription factors, such as IE180, EP0 and US1 proteins. The viral mRNAs have been divided into three temporal classes: immediate early (IE), early (E), and late (L). Immediately after the penetration of the virus to the host cell, the only PRV IE gene, the *ie180* gene is initiated to be expressed. This gene encodes the main transcription activator, which controls the expression of the rest of the PRV genes. Early genes are involved in the replication of the viral DNA, while the majority of the late genes code for structural elements of the virus. The PRV transcriptome has been characterized earlier with both short-read Illumina sequencing (Oláh et al., 2015) and long-read Pacific Biosciences (PacBio) RS-II sequencing (Tombácz et al., 2016) in our laboratory. The transcriptional dynamics have also been analyzed by multi-time-point real-time RT-PCR (Tombácz et al., 2009) and PacBio isoform sequencing (Tombácz et al., 2017a).

The existence of short upstream ORFs (uORFs) were demonstrated in a variety of organisms (Imai et al., 2006; Wen et al., 2009), including herpesviruses (Kronstad et al., 2013), but have not yet been identified in PRV. The uORFs exert their regulatory effect during translation through ribosome skipping, or through blocking the movement of the ribosomes by other ribosomes arrested on uORFs, or through the inefficiency of translational re-initiation (Hinnebusch et al., 2016). According to Calvo et al. (2009), the following four main characteristics are the most important in the translational efficiency of uORFs: a strong surrounding context of the AUG, which makes the recognition possible for the scanning ribosome subunit; evolutionary conservation; increased distance from the 5′ Cap of the mRNA, and finally the presence of multiple uORFs in the 5′ UTR.

Second- and third-generation sequencing platforms have proved to be extremely effective in describing the structural diversity of the transcripts and the dynamics of gene expression.

The Illumina platform has a high base accuracy and coverage, which renders it a great tool for the identification of transcriptional start sites (TSSs), transcriptional end sites (TESs), splice junctions, and RNA editing (Picardi et al., 2010; Oláh et al., 2015). However, the short read lengths lack the information needed for whole transcript assembly, and therefore it is difficult to identify the alternative TSSs, TESs, and splice isoforms. Long-read sequencing platforms developed by PacBio and Oxford Nanopore Technologies (ONT) brought the possibility of sequencing full-length cDNA and RNA molecules at the price of a lower throughput and higher sequencing error rates, especially in the ONT approach (Laver et al., 2015; Rhoads and Au, 2015). Long-read sequencing is superior to the short-read method for determining 5′ and 3′ UTR variants, splice isoforms, long non-coding transcripts, while they are especially effective for the detection of overlapping and embedded transcripts (Irimia et al., 2014; Tombácz et al., 2016, 2017b; Balázs et al., 2017). Although the ONT technique works with a high error rate, this does not a present a major problem in the case of high coverage and for well-annotated genomes. The major advantage of the ONT platform is that it can read nucleic acid sequences within the range of 200–800 bp, for which the PacBio and Illumina platforms are insensitive. Additionally, the ONT platform can be used for direct RNA sequencing that preserves read orientation and allows the detection of RNA modifications (Smith et al., in review).

Single-molecule sequencing approaches allow the reliable analysis of the expression dynamics of the viral genes and the total genome by inferring the relative amount of transcripts from the sequencing data (Oikonomopoulos et al., 2016; Tombácz et al., 2017a). PacBio reads have a shorter mean length than ONT reads but the former technique has an 11.7-fold higher sequencing accuracy.

Besides the above-mentioned innate drawbacks of next-generation sequencing, library preparation itself can also give rise to artifacts. The 5′ truncated reads can be a result of RNA degradation or incomplete reverse transcription, which impede the identification of TSSs. The tendency of the reverse transcriptase enzyme for skipping stretches of RNA between repeats, called template switching, causes artifactual introns or chimeric reads (Luo and Taylor, 1990; Cocquet et al., 2006).

In this study, we used the PacBio RS II and the ONT MinION sequencing platforms for the analysis of the PRV transcriptome. We also used data from our previous PacBio and Illumina sequencing studies for which the reasons as follows. We used these data for the confirmation of the new sequencing data and for the comparison of the obtained results. Additionally, our former studies identified low-abundance transcripts, but we had not published these earlier because they were represented by only a few sequencing reads. However, if these transcripts were have been detected in this current study, we considered it as a confirmation for their existence. See details in Section “Analysis of the PRV Transcriptome with Multiple Sequencing Techniques” for the design of the study. In this work, we did not use the first-generation Sanger sequencing, which can be useful for validation purposes in *de novo* sequencing of both genomic and cDNA sequences in case of low coverage of sequencing data due to the high accuracy of this technique. In our study, we did not need high accuracy – although we had it in Illumina and PacBio sequencing – because we have already sequenced and annotated the PRV genome (Tombácz et al., 2014) to which the sequencing reads were mapped. Further validation of the transcript identity is unnecessary because even the ONT, the least accurate technique alone is capable of identifying any transcripts with 100 per cent certainty by mapping them to the PRV DNA.

## Materials and Methods

### Cells and Viral Infection

Strain Kaplan of PRV was propagated on immortalized porcine kidney epithelial cell line (PK-15; ATCC CCL-33). Cells were cultivated in Dulbecco’s Modified Eagle’s Medium (Thermo Fisher Scientific) supplemented with 5% fetal bovine serum (Gibco Invitrogen) with 80 μg gentamycin/ml (Gibco Invitrogen) at 37°C, and in an atmosphere of 5% CO_2_. For the current experiments two biological replicates were conducted, while previous data was obtained from 17 separate biological replicates, 16 from previous PacBio sequencings and a single one from the Illumina sequencing.

### RNA Purification

*Total RNA isolation* was conducted using the Nucleospin RNA Kit (Macherey-Nagel) according to the manufacturer’s guidance. In short, infected cells were collected by centrifugation and then disrupted by the addition of lysis buffer (derived from the kit). Genomic DNA was digested by treating with RNase-free rDNase solution (supplied with the kit). Samples were eluted in a total volume of 50 μl nuclease free water. The potential residual DNA contamination was eliminated by treating with TURBO DNA-free Kit (Thermo Fisher Scientific). The RNA concentration was measured using a Qubit 2.0 Fluorometer through use of the Qubit RNA BR Assay Kit (Thermo Fisher Scientific).

*Poly(A)^+^ purification* The poly(A)^+^ RNA fraction was isolated from the samples using the Oligotex mRNA Mini Kit (Qiagen). The RNA samples were stored at -80°C until use.

*5′-Cap selection* Full-length cDNA synthesis was carried out by using the TeloPrime Full-Length cDNA Amplification Kit (Lexogen). Two microgram of total RNA was used for first strand cDNA synthesis according to the manufacturer’s recommendations. Base-pairing and ligation of the 5′ adapter to the DNA-RNA hybrid was carried out overnight at 25°C, followed by second strand synthesis using reagents supplied in the kit. Endpoint PCR was performed with the TeloPrime Kit. Each step was followed by purification on silica columns provided in the kit. Sample concentration was determined using a Qubit 2.0 Fluorometer through use of the Qubit (ds)DNA HS Assay Kit (Thermo Fisher Scientific).

### Testing for DNA Contamination

Two-step RT-qPCR was used to test for DNA contamination. First strand cDNA synthesis was performed using 70 ng of total RNA for each biological replicate, 2 pmol of the gene specific primer UL43_fw (CTGGTGCAGGCGTACGTGA), 0.25 μl of dNTP mix (10 μM final concentration), 1 μl of 5 × First-Strand Buffer and 0.25 μl (50 units/μl) SuperScript IV Reverse Transcriptase (Thermo Fisher Scientific). RT controls were used by replacing the RT enzyme with nuclease-free water in both biological replicates. RT-qPCR reaction was carried out in a total volume of 20 μl containing cDNA or RT control, both of the gene-specific primers UL43_fw, UL43_rev (GGATTTAATGCTAGTGGCGCA), and ABsolute QPCR SYBR Green Mix (Thermo Fisher Scientific) according to the manufacturer’s recommendation. The running conditions were as follows: 15 min at 95°C, followed by 35 cycles of 94°C for 25 s (denaturation), 60°C for 25 s (annealing), and 72°C for 6 s (extension). A DNA control was produced by replacing the cDNA with 6 ng of genomic DNA and nuclease-free water. 12% acrylamide gel electrophoresis and GeneRuler Ultra Low Range DNA Ladder (Thermo Fisher Scientific) was used for visualizing the amplicons. Staining was performed with GelRed (Biotium).

### Oxford Nanopore MinION Sequencing

*The ‘strand switching cDNA by ligation’ approach* Library from total mRNA was prepared using the Ligation Sequencing kit (SQK-LSK108; Oxford Nanopore Technologies) following the 1D Strand switching cDNA by ligation protocol. Briefly: (ss)cDNA synthesis was carried out using SuperScript IV Reverse Transcriptase (Thermo Fisher Scientific) and an anchored adapter-primer with (VN)T_20_ nucleotides (nts). A 5′ adapter sequence with three *O*-methyl-guanine RNA bases was added for the facilitation of strand switching. PCR was carried out using Kapa HiFi DNA polymerase (Kapa Biosystems) and the primers supplied in the kit. End repair was conducted using NEBNext End repair/dA-tailing Module (New England Biolabs) followed by adapter ligation using adapters (supplied in the kit) and NEB Blunt/TA Ligase Master Mix (New England Biolabs). The cDNA sample was purified between each step using Agencourt AMPure XP magnetic beads (Beckman Coulter) and the library concentration was determined using a Qubit 2.0 Fluorometer through use of the Qubit (ds)DNA HS Assay Kit (Thermo Fisher Scientific). Samples were loaded on R9.4 SpotON Flow Cells, and base calling was performed using Albacore v1.2.6.

The Cap-selected mRNA sample was subjected to end repair and adapter ligation steps – as was described above – before loading on the Flow Cells.

*The direct RNA sequencing approach* Libraries were prepared using the Direct RNA Sequencing Kit (SQK-RNA001; Oxford Nanopore Technologies) The first strand cDNA was synthesized by SuperScript IV Reverse Transcriptase (Thermo Fisher Scientific) using an RT adapter with T_10_ nts. Adapters, supplied in the kit, were ligated using T4 DNA ligase (New England Biolabs). The RNA-DNA hybrid was purified between each step by using Agencourt AMPure XP magnetic beads (Beckman Coulter), treated with RNaseOUT Recombinant Ribonuclease Inhibitor (Thermo Fisher Scientific). Sample concentration was determined using a Qubit 2.0 Fluorometer and Qubit DNA HS Assay Kit (Thermo Fisher Scientific). Libraries were loaded on R9.4 SpotON Flow Cells. Albacore software (v1.2.6) was used for base calling.

### PacBio RSII Isoform Sequencing

The preparation of cDNA samples was done according to the PacBio Isoform Sequencing (Iso-Seq) protocol using the Clontech SMARTer PCR cDNA Synthesis Kit. Single-stranded cDNAs were synthesized from the polyA+ RNAs by using 3′ SMART^®^ CDS Primer II A (included in the Clontech kit) or adapter-linked GC-rich random primers. The first-strand cDNA samples were amplified by PCR, using the SMARTer Kit and KAPA HiFi Enzyme (Kapa Biosystems) following the PacBio’s protocol. Five-hundred ng of cDNA was used for the SMRTbell library preparation, using the PacBio DNA Template Prep Kit 1.0. DNA/Polymerase Binding Kit P6 was used for the production of the polymerase/template complexes. Sequencing was carried out on an RSII sequencer with DNA Sequencing Reagent Kit 4.0. The movie lengths were 240 or 360 min (one movie was recorded for each SMRT cell). Base calling was performed and ROIs were generated using SMRT Analysis v2.3.0.

### Illumina HiScanSQ Sequencing

Illumina HiScanSQ platform was also used for PRV transcriptome sequencing. Briefly, a random-primed sequencing library was constructed with ScriptSeq v2 RNA-Seq Library Preparation Kit (Epicentre) for paired-end sequencing, and a single-end library was created for single-end sequencing, using oligo(VN)T20 primer.

### Transcript Annotation, Visualization, and *in Silico* Analysis

Reads were aligned to the genome of PRV strain Kaplan (KJ717942.1) and swine (*Sscrofa10.2*). Tophat v2.09 (Trapnell et al., 2009) and Bowtie (Langmead and Salzberg, 2012) software were used for the alignment of the Illumina reads, while for mapping the MinION and PacBio reads, we chose the GMAP v2017-04-24 (Wu and Watanabe, 2005) software, which produces the highest alignment rates along with an optimal hardware usage for RNAseq data (Križanović et al., 2017). For the PacBio platform reads with a mismatch or in-del ratio >5% were considered low quality and discarded from the analysis. The orientation of the reads was determined based on the presence of a 5′ adapter and the poly(A) tail. The known sequence of the 5′ adapter (PacBio: AGAGTACATGGG) and 40 A nucleotides were aligned to the soft clipped regions of reads in an interval of -10 bp to +30 bp from the start of the soft clip, using the Smith–Waterman algorithm. Alignment score parameters were as follows: match = +2, mismatch = -3. Alignment score thresholds were set to 24 for the poly(A) tail and 18 for the 5′ adapter sequence. For the ONT datasets the same algorithm was applied with the following parameters: match = +2, mismatch = -3, while for the poly(A) tail a threshold of 20 for cDNA and 13 for direct RNA was set. The 5′ adapters could not be determined *in silico*. To eliminate false transcripts originating from strand switching or from possible DNA contamination, reads with a poly(A) tail or a 5′ adapter sequence on both sides of the read, or without a poly(A) tail were discarded.

The validation criteria for the identification of novel TES and TSS positions were as follows. The last position before a poly(A) tail was considered the TES if the reference genome contained less than 3 (A)s at the 3′ end of the read, and if at least two reads from libraries with separate reverse transcription reactions had their 3′ end at the exact position. Every other poly(A) tail was considered an artifact, and the read containing it was not used for TES determination. A certain TSS was accepted if we found at least two independent sequencing reads from two different libraries with a maximum of five-nucleotide variation.

Reads with a greater than 10 nt difference in their 5′ or 3′ ends were considered new length isoforms only if their end coordinates matched in case of at least two sequenced libraries with separate RTs (L: longer 5′ UTR, S: shorter 5′ UTR, AT: alternative termination). New length isoforms harboring a different open reading frame (ORF) from that of the known transcript were considered a putative new coding transcript. New length isoforms lacking an ORF were named non-coding transcripts (nc: non-coding). Reads mapping to a known transcript but with opposite direction were named antisense transcripts (AS: antisense). Long reads spanning at least two known transcripts with different directions were named complex transcripts (C: complex). The read quality score was defined as the cumulative number of substitutions, insertions and deletions divided by the number of nucleotides sequenced.

**FIGURE 1:**
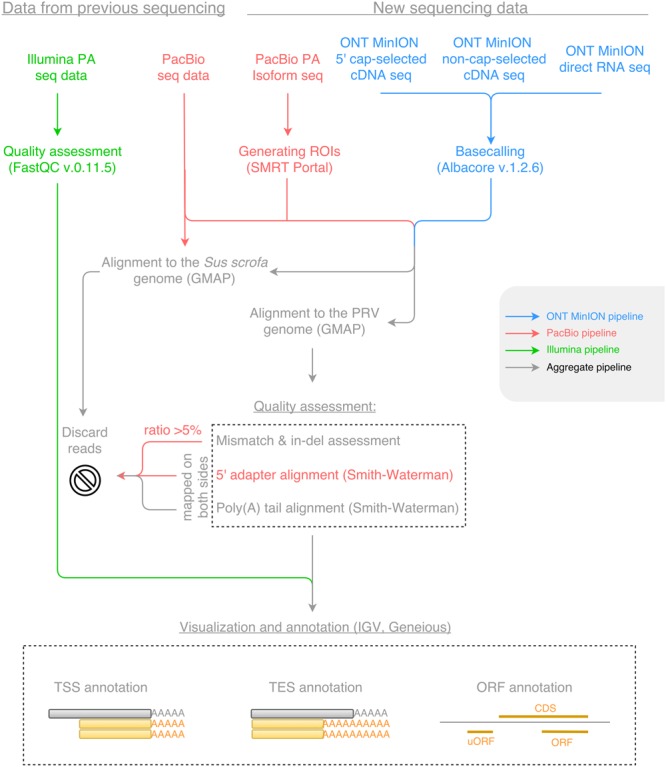
The workflow of data analysis for Illumina, Pacific Biosciences (PacBio) and Oxford Nanopore Technologies (ONT) MinION sequencing datasets. Blue color represents the pipeline for the ONT MinION sequencing data; red color represents the pipeline for the PacBio sequencing data; green color represents the pipeline for the Illumina sequencing data; while gray color represents the aggregate pipeline of all sequencing data.

Open reading frames were determined using the Geneious program suit (Kearse et al., 2012), and the average length of the ORFs constrained between the START and STOP codon of each known coding sequence (CDS) was calculated for each reading frame, where frame +1 was the frame of the CDS. The uORFs were defined as starting with upstream AUGs preceding the canonical ORFs either with a STOP codon upstream or downstream of the AUG of the canonical ORF. The Kozak consensus sequence (GCCRCCATGG) was determined *in silico* using the Smith–Waterman algorithm with a gap opening score of -10, a gap extension score of -1 and a filtering minimal score of 80%. Workflows for sequencing data are shown in **Figure 1**. The Geneious R10, and the IGV v2 (Thorvaldsdóttir et al., 2013) programs were used for transcript annotation and visualization.

## Results

### Analysis of the PRV Transcriptome with Multiple Sequencing Techniques

**FIGURE 2:**
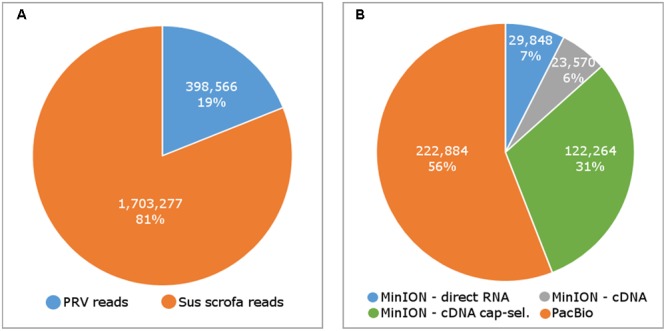
The number of sequencing reads mapping to the pseudorabies virus (PRV) and host (Sus scrofa) genome **(A)**, and the number of reads by sequencing methods **(B)**.

**FIGURE 3:**
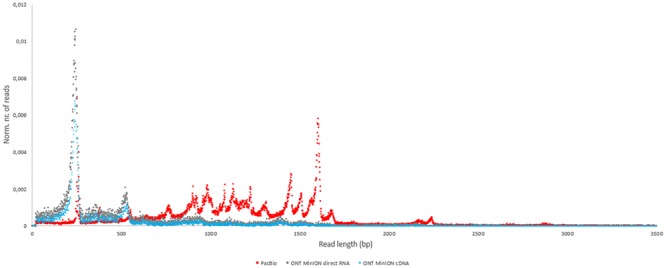
The length distribution of reads for each sequencing method. The number of reads was normalized with the total number of reads for each sequencing platform (red dots: PacBio; blue dots: ONT MinION cDNA; gray dots: ONT MinION direct RNA).

In this study, we carried out ONT MinION and PacBio RSII sequencings of the PRV transcriptome. Additionally, we re-evaluated the data obtained in our recent publications using Illumina HiScanSQ (Oláh et al., 2015) and PacBio isoform sequencing (Tombácz et al., 2016) in light of the novel results. Amplified methods were used in each case, except the RS-II technique, where we also used a non-amplified isoform sequencing approach and the ONT direct RNA sequencing method, which is also a non-amplified technique. We applied oligo(dT)-based reverse transcription (RT) in all protocols except the random primer-based technique used in PacBio and Illumina sequencing. The strand switching cDNA by ligation sequencing of ONT MinION technique yielded 23,570 reads with an average read length of 769 bp and an average read depth of 75, while the direct RNA sequencing yielded 29,848 reads with an average read length of 909 bp and average read depth of 162 (**Figure 2**). The PacBio RSII long read sequencing yielded a total of 222,884 reads with an average read length of 1,337 bp and average read depth of 909. The Illumina HiScanSQ platform had a quality score of 34,42. The percentage of INDELs and mismatches of the mapped reads for the PacBio RSII and ONT sequencing data is listed in **Table 1**. The PacBio RSII platform yielded transcripts with a median length of 1,271; transcripts identified by MinION cDNA sequencing had a median read length of 510 bp; while the MinION direct RNA sequencing median read length was 837 bp. These results show the suitability of the MinION platform for finding relatively small full-length transcripts and transcript isoforms. The read length distribution for each sequencing technique is shown in **Figure 3**.

**Table 1:** The percentage of mismatches, insertions and deletions for the ONT MinION cDNA, direct RNA, and the PacBio sequencing platforms.

	Mismatch	Insertion	Deletion
	(%)	(%)	(%)
PacBio RSII	0.55	0.86	1.16
ONT MinION cDNA by ligation	8.13	3.23	6.42
ONT MinION direct RNA	8.1	3.05	9.27

MinION sequencing of the 5′ Cap-selected RNAs resulted in 122,264 reads mapping to the PRV genome with an average aligned read length of 362 bp and average read depth of 333. In contrast to the fragmented PRV reads, we obtained full-length reads for the host cell transcripts. We assume that the short read length was due to the incomplete reverse transcription caused by high GC-content of the PRV mRNAs (Shi and Jarvis, 2006).

Using three sequencing platforms two of which are capable of long-read sequencing, we confirmed the ends of all previously described transcripts, and in addition, we precisely determined the 5′ and 3′ termini of 91 novel transcripts. The number of novel transcripts and the platform on which they were identified is listed in Supplementary Table 1.

**FIGURE 4:**
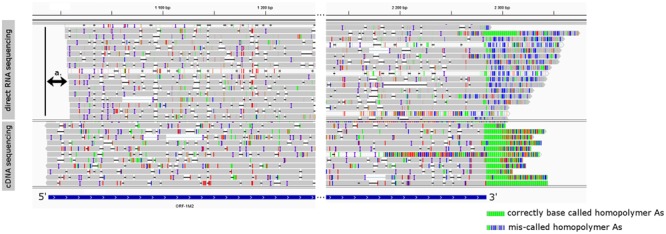
The visual comparison of MinION direct RNA sequencing data with the MinION cDNA sequencing data. The gray rectangles are reads mapped to the genome of PRV representing transcript orf-1M2. Colored lines represent mismatch bases and INDELS. Reads were visualized using IGV, and their 5′ and 3′ region was cropped for better observation. The blue box represents the annotated orf-1M2 transcript. Double-arrow a. denotes the missing 5′ end of the MinION direct RNA sequencing data. On the 3′ end of the reads green lines represent homopolymer As in the correctly base called poly(A) tail, while the blue-gray lines in the MinION direct RNA sequencing represent the miscalled nucleotides of the poly(A) tail.

We observed that the direct RNA sequencing resulted in poor 5′ and 3′ read ends. The expected TSS of already known transcripts were missing on the 5′ ends of the reads, which were 23 bp shorter on average (**Figure 4**), and only 36 of the total reads were carrying poly(A) tails, while most of the reads showed a CT-rich region downstream from their TES (**Figure 4**). Both shortcomings are the result of the current technical limitations of the ONT MinION direct RNA sequencing method. The missing nucleotides at the 5′ end of the reads are probably caused by the premature release of the mRNA molecule by the motor protein, which controls the speed of the progress of RNA molecules through the nanopores. This may result in the rapid transition of the RNAs which can perturb the base calling of the region. Missing poly(A) tails are assumed to be the result of the direct RNA base caller script miscalling the nucleotides of the DNA adapter ligated downstream of the poly(A) tail, and the DNA signal muddles the raw signal of the downstream ‘A’ homopolymer.

No residual DNA was present in our sequencing libraries according to our test for DNA contamination. A single band appeared in the 12% polyacrylamide gel electrophoresis for both the PacBio and the MinION sequencing libraries and the gDNA control, representing a 51-bp long amplicon. No bands were shown in RT controls (Supplementary Figure 1).

### Novel Putative Protein-Coding Genes

**FIGURE 5:**
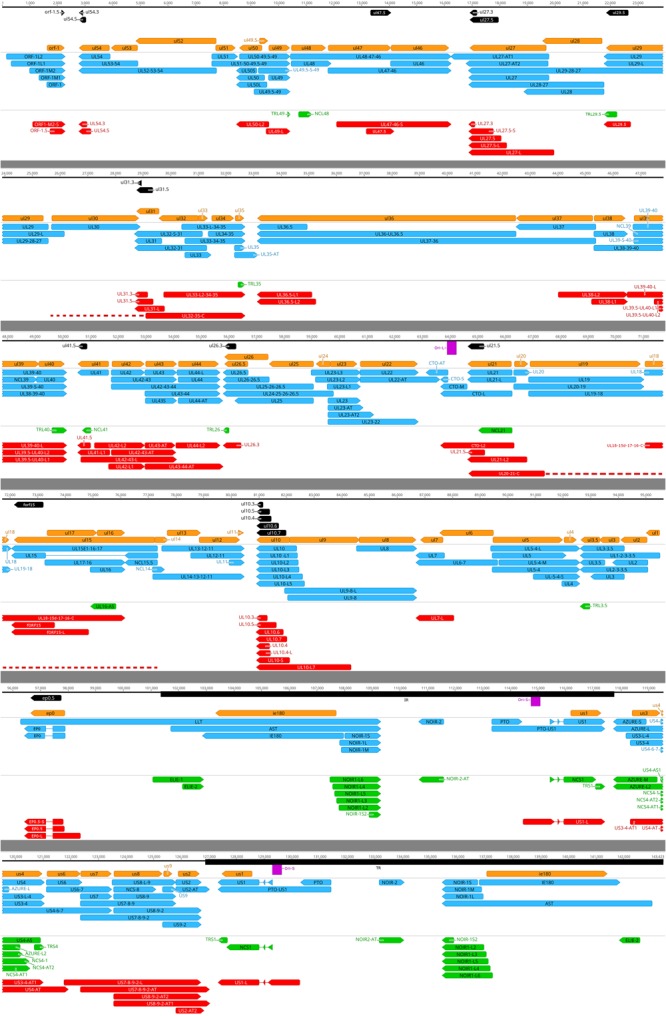
Annotation of the PRV transcriptome. Coding sequences (CDS): orange arrow-rectangles; novel putative ORFs: black arrow-rectangles; already known transcript isoforms: blue arrow-rectangles; novel non-coding transcripts: green arrow rectangles; novel protein coding transcripts and transcript isoforms: red arrow-rectangles.

All of the putative protein-coding transcripts identified in this work are truncated forms of longer host genes into which they are embedded. The novel ORF-containing transcripts can be categorized on the basis of whether the ORF is positioned in in-frame (tORFs), or is frame-shifted (f)ORFs compared to the ORF of the host gene. Our investigation revealed altogether 18 transcripts with tORFs and a single transcript with fORF, the latter being located within the fORF15 transcript (**Figure 5** and Supplementary Table 2). The tORFs were marked 0.7 to 0.2, indicating their size compared to the already known 0.5 variants. The highly expressed *ul10* gene is notable because it produces a large number of coding transcripts and transcript isoforms. In addition to the previously annotated five length isoforms of UL10 transcripts, we detected an additional eight variants of this transcript among which five bear distinct tORFs. We detected a truncated version of the *orf1* gene encoding the membrane protein pUL56 (Daniel et al., 2016). The *orf1.5* gene harbors a short 102-bp long ORF. While no full-length monocistronic transcript from *ul47* gene has been detected until now, we could identify a truncated RNA from this genomic region encoded by a putative gene (*ul47.5*) embedded into *ul47* gene. The 5′ Cap-selected sequencing data helped confirm the 5′ ends of these transcripts, including UL54.3, UL27.3, and UL31.3. Additionally, we also describe the discovery of a truncated form of the *ep0* gene (*ep0.5*), which is an important transcription factor of PRV. The fORFs are suggested to represent alternative coding potential (Normark et al., 1983; Vanderperre et al., 2013). However, because the sequences of fORFs are constrained by the sequences of the overlapping functional gene and thereby would pose an extreme challenge for the natural selection, we therefore think that these ORF-containing transcripts are non-coding. These short embedded transcripts may have been overlooked by Northern blot analysis because of their relatively low abundance. These transcripts have not been identified by both the Illumina and the PacBio sequencing because their sizes are within a range, which is not optimal for these techniques. The ONT MinION sequencing proved extremely helpful in the detection of full-length transcripts with less than 800 bp size.

### Novel Non-coding Transcripts

We detected a total of 19 novel non-coding (nc)RNAs (Supplementary Table 2). The transcription of embedded non-coding transcripts can start from the same promoter as the longer transcripts of the host genes. These 3′-truncated ncRNAs are named as “NCL” if they are in the UL region of the virus, or “NCS” if “*us*” genes are the hosts. The 5′-truncated transcripts are supposed to have their own promoters but have no or no in-frame ORFs. These transcripts are termed “TRL” if their hosts are “*ul*” genes or “TRS” if they are embedded in any of the “*us*” genes. Transcripts with a length larger than 200 bp are termed as long non-coding (lnc)RNAs, while the smaller transcripts are called short non-coding (snc)RNAs. TRL49 with its 93 bp length is the shortest non-coding transcript of PRV and belongs to the latter category. The rest of the newly discovered transcripts are all lncRNAs. It has been shown in our earlier publication (Tombácz et al., 2016) that the upstream domain of *ul15* gene (NCL15) can be expressed separately; here we show that the downstream domain is also transcribed independently from the full-length *ul15* gene. This lncRNA is named as fORF15 because it contains an out-of-frame ORF (**Figure 4**). We also identified two novel antisense RNAs: UL16-AS overlapping the UL16 mRNA and US4-AS overlapping the US4 mRNA. Two novel ncRNAs (ELIE-1 and -2: ***e****mbedded in* ***L****LT and overlap* ***IE****180*) overlapping the 3′-end of *ie180* gene in a tail-to-tail manner have also been detected. The longer transcript (ELIE-1) is initiated at the UL region, while the TSS of the shorter transcript (ELIE-2) is located at the IR region and therefore its gene is represented in two copies in the PRV genome.

### TSS and TES Isoforms

Transcription start site isoforms differ from each other in the length of their 5′ UTRs. We identified altogether 39 novel TSS variants (Supplementary Table 3). Putative TATA boxes were detected *in silico* for five TSS isoforms; they were mapped on average 33.5 nt upstream from the TSS. We assume that the rest of the transcripts are expressed from TATA-less promoters, which is a common phenomenon in eukaryotes (Yang et al., 2007). The low expression level of these alternative transcripts may be explained by their control by unconventional promoters. TSS isoforms were named by adding the “S” letter tag if they are shorter than the annotated or most common isoform or an “L” tag if they are longer than the common variant. We termed transcripts with alternative TES by adding the two-letter suffix “AT” to the name. The putative poly(A) signals (PA-signal) were found to be located at an average distance of 20.52 nt from the poly(A) sites (PAS). In many cases, only the longer transcript variant forms an overlap with the adjacent gene. The longest isoform (L7) of UL10 transcripts almost entirely overlaps the divergent *ul9* gene, therefore it can also be considered a complex transcript. The *ul41-44* genomic region exhibits an especially complex transcription pattern including a variety co-transcription combination (producing mono-, bi-, and tricistronic transcripts) and a large polymorphism in the alternative 5′ and 3′ ends of the transcripts. The transcripts expressed from the embedded genes also exhibit variation. We also identified novel transcripts overlapping the replication origins of the virus: CTO-L2 overlaps the Ori-L and US1-L overlaps both Ori-Ss.

### Novel Complex Transcripts

Three novel complex transcripts (UL32-35-C, UL20-21-C, and ul18-15d-17-16-C) were detected using the ONT MinION direct RNA sequencing method, each being confirmed by the PacBio RSII platform. The TSS of UL32-35-C and UL20-21-C could not be precisely determined; therefore, we illustrated them as if they were controlled by the promoter of the closest upstream gene standing in the same orientation as the transcripts (**Figure 5**, dashed line).

### Novel Transcriptional Overlaps on the PRV Genome

**FIGURE 6:**
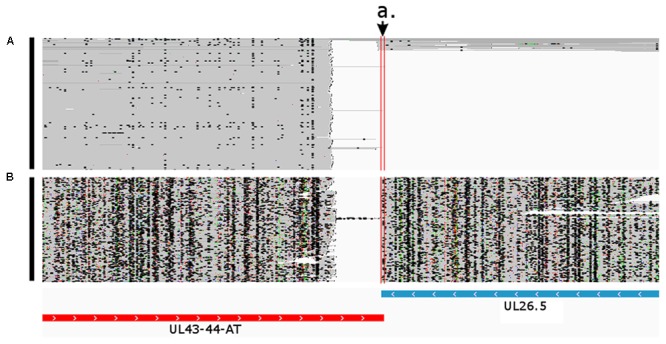
Convergent transcriptional overlap of UL43-44-AT and UL26.5. The arrow a. points to the overlapping region delimited by the vertical red lines. The blue box represents the already known UL26.5 transcript, while the red box represents the novel UL43-44-AT transcript. Reads of the PacBio IsoSeq **(A)** and MinION Direct RNA **(B)** sequencing were visualized in compact mode using IGV.

**FIGURE 7:**
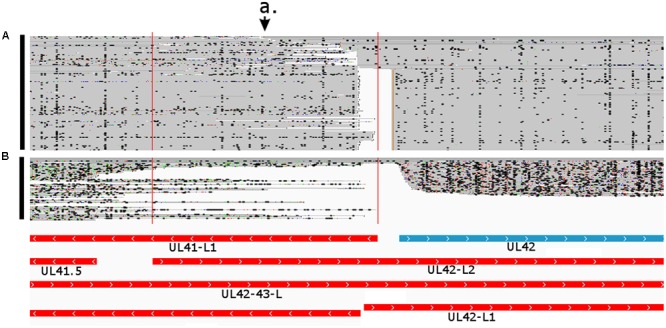
Divergent transcriptional overlap of multiple transcripts in the ul41-ul43 gene cluster. The arrow a. points to the overlapping region delimited by the vertical red lines. The blue box represent already known UL42 transcript, while the red boxes represent novel transcripts. Reads of the PacBio IsoSeq **(A)** and MinION cDNA **(B)** sequencing were visualized in compact mode using IGV.

**FIGURE 8:**
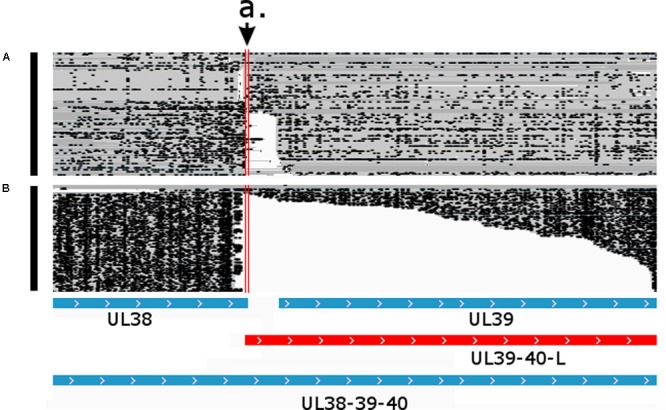
Parallel transcriptional overlap of the UL38 and UL39-40-L transcripts. The arrow a. points to the overlapping region delimited by the vertical red lines. Blue boxes represent already known transcripts, while the red box represents the novel UL39-40-L transcript. Reads of the PacBio IsoSeq **(A)** and MinION Direct RNA **(B)** sequencing were visualized in compact mode using IGV.

An overlap can be ‘hard’ if there are no non-overlapping transcripts, or they can be ‘soft’ if only the longer transcripts overlap each other. In the latter case, the longer isoforms are generally expressed in a low abundance. In this work, we identified 121 novel transcriptional overlaps, among which 40 stand in a head-to-head (divergent; 5′ to 5′-end; **Figure 6**), 70 in a tail-to-head (parallel; 5′ to 3′-end; **Figure 7**), and 11 in a tail-to-tail (convergent; 3′ to 3′-end; **Figure 8**) orientation (Supplementary Table 4). The sizes of the novel overlapping regions range from 5 to 4,349 bp. The transcription of the longer versions of UL49 and UL50 transcripts produce an overlap between the two RNA molecules. The newly identified UL50-L2 is longer than the previously described UL50-L, which produces an increased extent of overlap with its neighbor *ul49* gene. We detected four divergent overlaps between the previously known and newly discovered UL41 isoforms and UL42 isoforms. The fORF15 and its longer isoform fORF15-L overlap in a divergent manner with UL17-16. We also found that a tail-to-tail overlap was formed between the UL43-44-AT and the convergent transcript isoforms of UL26 transcripts, and between the UL30 and UL31.5/UL31.3 transcripts. Head-to-tail overlaps occur between the genes of a polycistronic unit: the upstream genes of a polycistronic transcript always overlap transcriptionally with the downstream genes. The smaller embedded genes also overlap with the larger host gene.

### Upstream ORFs

Using *in silico* analysis, we determined 145 uORFs both in the previously identified and the novel TSS isoforms of the transcripts (Supplementary Figure 2 and Supplementary Table 5). The mean uORF length was 650.92 nt (*SD* = 620.64 bp), the smallest being just 9 bp, while the longest being 2,532 bp. Three of the uORFs present a strong uATG context by bearing a Kozak consensus sequence. The mean distance of the ATG from the 5′ end of the transcript is 900.93 bp (*SD* = 727.39 bp), the smallest distance being only 2 bp in ul50-L-2 while the longest being 2,596 bp in ul9-8-L.

### Confirmation of Splicing with Direct RNA Sequencing

It is well-known that reverse transcription produces false identification of splicing (Cocquet et al., 2006) due to the phenomenon of template switch at the repetitive sequences of the RNA molecules. To validate our earlier results, we carried out direct RNA sequencing. This technique helped us to confirm the occurrence of splicing in UL15, two isoforms of EP0, and US1 PRV transcripts.

## Discussion

Both short-read and long-read sequencing have become popular tools for structural and functional analysis of the global transcriptomes (Mortazavi et al., 2008; Wang et al., 2009). Both approaches have their advantages and drawbacks. While producing a massive amount of accurate read data, the Illumina short-read sequencing technique is not optimal for annotating polycistronic transcripts or UTR and splice isoforms (Steijger et al., 2013). The PacBio and ONT cDNA sequencing solves this problem by generating long (>200 bp) reads, with the latter being capable of sequencing up to 60 kb amplicons (Madoui et al., 2015). Using ONT sequencing, we were able to detect transcripts within the range of 200–800 bp, which are not optimal for either the PacBio or the Illumina systems. By using direct RNA sequencing, we were able to circumvent the generation of artifactual splice isoforms caused by the phenomenon of template switching (Cocquet et al., 2006).

One of the major aims of this study was to complete the transcriptional landscape of the PRV. We also intended to investigate the extent of transcriptional overlaps, which we believe to play an important role in the control of the global gene expression of the viruses. Another purpose of our present work was to demonstrate the utility of two long-read sequencing techniques for the discovery of novel genes, transcripts and transcripts isoforms using a herpesvirus as a model. These methods would also be useful for the same purpose in other viral families. The novel transcripts were only accepted if we could detect them in multiple independent sequencing reads many of them produced by different sequencing platforms.

Our multi-platform approach identified 91 novel RNA molecules, including putative protein coding and non-coding transcripts, as well as novel 5′ and 3′ UTR isoforms that have not previously been annotated, probably because of their short length and low abundance. We also detected very long RNA molecules containing genes in opposite orientation relative to each other, named complex transcripts. This is consistent with earlier studies based on Real-time RT-PCR analysis (Tombácz et al., 2016), which demonstrated that almost the entire PRV genome exhibits varying levels of antisense transcriptional activity. We assume that these complex transcripts are the source for some part of the antisense transcript fragments, which were detected as chimeric reads (data not shown). Another possibility is that the antisense transcripts are produced from their own promoters such as the LAT promoter controlling the expression of long-latency transcript in PRV (Markovitz et al., 1999). Additionally, we confirmed or corrected the nucleotide sequence, as well as the precise TSS and TES positions of already annotated transcripts with base-pair precision.

Using *in silico* analysis, we predicted potential uORFs present on the longer 5′ UTR isoforms. These uORFs may regulate the translation of their neighboring CDS in PRV in the same manner as described in eukaryotes or in Herpes simplex virus (Markovitz et al., 1999).

Long-read cDNA sequencing often produces a large number deleted region within the nucleic acid sequences mainly due to template switching. However, using direct RNA sequencing technique, we detected a low number of real splicing events in the PRV transcriptome. It is possible that the relatively low throughput of the current direct RNA sequencing technology hinders the discovery of rare splice variants, which may be detected with other techniques using amplified libraries. In this study, we were able to confirm the existence of three splicing events (in the Ul15, EP0, and US1 transcripts) with a novel technique (direct RNA sequencing, ONT), which we had detected in our earlier publication (Cocquet et al., 2006).

In this work, a complex meshwork of transcriptional overlaps was identified. Our analysis enriched the number of known parallel, convergent and divergent transcriptional overlaps between adjacent and distal genes in the PRV transcriptome. For example, this study revealed a complex transcriptional landscape around the *ie180* gene: besides the gene-length convergent overlap between the IE180 mRNA and LLT/AST transcripts, the novel ELIE ncRNAs were found to form head-to-head overlaps, while the NOIR2 transcripts form a tail-to-tail overlap with the IE transcript. The *ie180* gene encodes the major transactivator protein of the PRV; its central role may explain why this gene is controlled by ncRNAs in such a complex manner.

In principle, the transcriptional overlaps may be the result of economical utilization of the viral DNA – however, the total gain would be minor. In our earlier study (Tombácz et al., 2016), we put forward another explanation, which is based on the interaction of transcriptional apparatuses at the transcriptionally overlapping regions. This mechanism has been proposed for individual genes pairs (Hobson et al., 2012; Pelechano and Steinmetz, 2013). Our Transcriptional Interference Network hypothesis claims that gene expressions are controlled by the activity of closely spaced genes in a system level (Boldogkői, 2012). Thus, it is possible that in certain cases not the transcripts, but the transcription itself is important. However, it does not mean that these are mutually exclusive. In this study, we also identified novel transcripts that overlap the replication origins of PRV. It was previously hypothesized that this form of overlaps play a role in the mutual control of replication and transcription by the interaction between the machineries of the two systems (Huvet et al., 2007; Tombácz et al., 2015).

### Data Deposition

The Illumina PA Seq datasets analyzed during the current study are available in the European Nucleotide Archive database accessible under accession: PRJEB9526.

The PacBio SMRT and Iso-Seq datasets analyzed during the current study are available in the European Nucleotide Archive PRJEB12867 and PRJEB17709.

The ONT MinION datasets analyzed during the current study are available in the Sequence Read Archive database data base accessible under accession: PRJNA417577.

Annotations are available in the FigShare database accessible under doi: 10.6084/m9.figshare.5593240 and doi: 10.6084/m9.figshare.5593243.

## Author Contributions

NM carried out the ONT MinION cDNA and direct RNA sequencing, analyzed the data, participated in the sequence alignment and drafted the manuscript. DT carried out the PacBio sequencing, the ONT MinION sequencing of the 5′ cap-selected transcripts, participated in the MinION direct RNA sequencing, in the design of the study, and took part in drafting the manuscript. AS participated in the sequence alignment and carried out the *in silico* analysis. ZC propagated the cells, prepared the RNA, DNA, and cDNA samples and participated in the MinION sequencing. MS participated in the coordination and design of the study. ZB conceived, designed and coordinated the study and wrote the manuscript. Funding acquisition: MS, ZB, and DT. All authors have read and approved the final version of the manuscript.

## Conflict of Interest Statement

The authors declare that the research was conducted in the absence of any commercial or financial relationships that could be construed as a potential conflict of interest.
