# Supplementary material for: Multi-Platform Sequencing Approach Reveals a Novel Transcriptome Profile in Pseudorabies Virus
Source: Front Microbiol. 2018 Jan 22;8:2708. doi: 10.3389/fmicb.2017.02708 (PMC5786565; doi:10.3389/fmicb.2017.02708)
Supplement: Supplementary file 1 [file Data_Sheet_1.docx]

Supplementary Material

**Multi-platform Sequencing Approach Reveals a Substantial Transcriptome Diversity in Pseudorabies Virus**

**Norbert Moldován^1†^, Dóra Tombácz^1†^, Attila Szűcs^1^, Zsolt Csabai^1^, Michael Snyder^2^, Zsolt Boldogkői^1*^**

^1^Department of Medical Biology, Faculty of Medicine, University of Szeged, Szeged, 6720, Hungary

^2^Department of Genetics, School of Medicine, Stanford University, Stanford, California, 94305, USA

^†^These authors contributed equally to this work.

**Correspondence:** Zsolt Boldogkői: [boldogkoi.zsolt@med.u-szeged.hu](mailto:boldogkoi.zsolt@med.u-szeged.hu)

**Supplementary figure 1. Testing for DNA contamination by RT-qPCR.**

**Panel I.1.** Acrylamide gel electrophoresis of the PCR products amplified from the ul43 gene segment. The templates used for the PCR analysis were as follows. D: PRV DNA control; lanes A and B: cDNAs from the PacBio or MinION library, respectively; lanes Ac and Bc: no-RT control of the A and B samples, respectively; lane M: DNA ladder (GeneRuler Ultra Low Range DNA Ladder; Thermo Fisher Scientific). Staining was performed with GelRed (Biotium). A 51-bp PCR product can be observed in lanes A, B and D, while no bands appear in lane Ac and Bc, which indicates that the sequencing libraries were free from DNA contamination.

**Panel I.2.** The running curves of the same samples as above using RT-qPCR. This figure also shows that the RNA solutions do not contain DNA contamination.

**Panel II.** Sequencing reads produced by PacBio and MinION sequencing were visualized by IGV. The sequencing reads are illustrated by gray bars. The green bars indicate the poly(A) tail. The blue bar with white arrows shows the UL43 transcripts, while the two red boxes indicate the positions of gene-specific primers UL43-fw and UL43-rev used for RT-qPCR. The presence of poly(A) tail on the reads indicate that the reads are originated from mRNA and not from DNA potentially contaminating the samples.

**Supplementary figure 2. The positions of putative upstream ORFs on the PRV genome.** Coding sequences (CDS): orange arrow-rectangles; novel putative ORFs: yellow arrow-rectangles; putative upstream ORFs (uORFs): black arrow rectangles; already known and novel transcript isoforms: grey arrow-rectangles.

**Supplementary Table 1.** **The number of novel transcript isoforms detected using different platforms.**

|  |  | **Non-coding** | |  |  |  |  |  |  |
| --- | --- | --- | --- | --- | --- | --- | --- | --- | --- |
|  | **Putative protein coding** | **Sense** | **Antisense** | **Complex** | **5'-L-Isoform** | **5'-M-Isoform** | **5'-S-Isoform** | **AT-isoform** | **Total by seq.:** |
| **ONT cDNA** | 3 | 2 |  |  | 1 |  | 1 |  | 7 |
| **ONT cDNA - PacBio IsoSeq** | 1 | 1 |  |  | 1 |  | 1 |  | 4 |
| **ONT cDNA - Illumina PA seq** |  | 3 |  |  |  |  |  |  | 3 |
| **ONT cDNA - ONT direct RNA - PacBio IsoSeq** |  |  |  |  |  |  |  |  | 0 |
| **ONT direct RNA** | 7 | 4 |  | 1 | 2 |  |  |  | 13 |
| **ONT direct RNA - PacBio IsoSeq** | 2 |  |  | 3 |  |  |  |  | 4 |
| **PacBio IsoSeq** | 6 | 7 | 2 |  | 27 | 1 | 4 | 9 | 57 |
| **PacBio IsoSeq - Illumina PA seq** |  |  |  |  |  |  |  | 3 | 3 |
| **Total by type:** | 19 | 17 | 2 | 4 | 31 | 1 | 6 | 12 | 91 |

**Supplementary Table 2.** **Novel putative coding and non-coding PRV transcripts.** Novel putative protein coding transcripts were termed .5 indicating a putative N-terminally truncated protein product, and .2 to .7 according to the ORF length variant present in a given transcript. Non-coding transcripts with an orientation identical with the mRNAs were labelled with an ‘NC’ prefix, while antisense non-coding transcripts with an ‘AS’ suffix. The long complex transcripts spanning multiple CDSs with different orientation were labelled with ‘C’ letters. The asterisk (*) next to a TSS indicates that the given TSS was confirmed by the 5’ Cap selection data. The ✓ (check mark) indicates the platform on which a given transcript isoform was detected.

| **Transcript description** | | | | | | **Confirmed on platform** | | | |
| --- | --- | --- | --- | --- | --- | --- | --- | --- | --- |
| **Transcript** | **TATA** | **TSS** | **PA Signal** | **TES** | **Orientation** | **Illumina PA** | **ONT cDNA** | **ONT dRNA** | **PacBio** |
| **ORF-1.5** |  | 1721 | 2257-2262 | 2284 | + |  |  |  | ✓ |
| **UL54.3** |  | 3063* | 2783-2788 | 2769 | - |  |  | ✓ |  |
| **UL54.5** |  | 3211 | 2783-2788 | 2769 | - |  |  | ✓ |  |
| **TRL49** |  | 10321* | 10389-10394 | 10414 | + |  |  | ✓ |  |
| **NCL48** |  | 10724 |  | 11197 | + |  |  | ✓ |  |
| **UL47.5** |  | 13169 |  | 14179 | + |  |  | ✓ |  |
| **UL27.3** |  | 17098* |  | 16869 | - |  |  | ✓ |  |
| **UL27.5** |  | 18052 |  | 16869 | - |  |  |  | ✓ |
| **TRL29.2** |  | 22235 |  | 21775 | - |  |  |  | ✓ |
| **UL29.2** |  | 22737 |  | 21775 | - |  |  | ✓ | ✓ |
| **UL31.3** |  | 29163* |  | 28692 | - |  |  | ✓ |  |
| **UL32-35-C** |  | 29093 | 32658-32663 | 32683 | + |  |  | ✓ | ✓ |
| **UL31.5** |  | 29365 |  | 28694 | - |  | ✓ |  |  |
| **TRL35** |  | 32432 | 32658-32663 | 32683 | + | ✓ | ✓ |  |  |
| **TRL40** |  | 49607 | 50117-50122 | 50136 | + |  | ✓ |  |  |
| **UL41.5** |  | 51010 |  | 50541 | - |  | ✓ |  |  |
| **NCL41** |  | 51012 |  | 50710 | - |  | ✓ |  | ✓ |
| **TRL26** |  | 56012* |  | 55794 | - |  |  | ✓ |  |
| **UL26.3** |  | 56456 |  | 55794 | - |  |  | ✓ | ✓ |
| **UL20-21-C** |  | 64679 |  | 67430 | - |  |  | ✓ | ✓ |
| **UL21.5** |  | 65265 |  | 64644 | - |  |  | ✓ |  |
| **NCL21** |  | 66258 |  | 65038 | - |  |  |  | ✓ |
| **UL18-15d-17-16-C** |  | 71069 | 76148-76153 | 76154 | + |  |  | ✓ |  |
| **fORF15** | 73566-73580 | 73610 |  | 72049 | - |  |  |  | ✓ |
| **UL16-AS** |  | 75826 |  | 74915 | - |  |  |  | ✓ |
| **UL10.3** |  | 81300 |  | 80899 | - |  | ✓ |  | ✓ |
| **UL10.4** |  | 81382 |  | 80906 | - |  | ✓ |  |  |
| **UL10.5** |  | 81621 |  | 80899 | - |  |  |  | ✓ |
| **UL10.6** |  | 81884 |  | 80899 | - |  |  |  | ✓ |
| **UL10.7** |  | 82006 |  | 80899 | - |  |  |  | ✓ |
| **TRL3.5** |  | 92969 |  | 92618 | - |  |  | ✓ |  |
| **EP0.5** |  | 97866 |  | 96421 | - |  |  |  | ✓ |
| **ELIE-1** |  | 101069 | 102872-102877 | 102896 | + |  |  |  | ✓ |
| **ELIE-2** |  | 102143 | 102872-102877 | 102896 | + |  |  |  | ✓ |
| **NOIR-1S2** |  | 108887 | 109278-109283 | 109304 | + |  | ✓ |  |  |
| **NCS1** | 115408-115422 | 115437 |  | 117094 | + | ✓ |  |  | ✓ |
| **TRS1** |  | 117074 | 117375-117380 | 117407 | + | ✓ | ✓ |  |  |
| **TRS4** |  | 120682 |  | 121045 | + | ✓ | ✓ |  |  |
| **US4-AS** |  | 120911 |  | 119427 | - |  |  |  | ✓ |
| **NCS4-1** | 119401-119415 | 119431 |  | 120249 | + |  |  |  | ✓ |

**Supplementary Table 3.** **Novel 5’ and 3’ UTR length isoforms of the PRV transcriptome**. The long 5’ UTR isoforms were labelled with adding an ‘L’ letter to the name of the transcripts, medium 5’ UTR isoforms were labelled with ‘M’, while the short 5’ UTR isoforms were labelled with ‘S’ suffixes. The 3’ UTR isoforms of novel alternatively terminating transcript were labelled with ‘AT’ suffixes. The ✓ (check mark) indicates the platform on which a given transcript isoform was detected.

| **Transcript description** | | | | | | **Confirmed on platform** | | | |
| --- | --- | --- | --- | --- | --- | --- | --- | --- | --- |
| **Transcript** | **TATA** | **TSS** | **PA Signal** | **TES** | **Orientation** | **Illumina PA** | **ONT cDNA** | **ONT dRNA** | **PacBio** |
| **ORF1-M2-S** |  | 1104 | 2257-2262 | 2284 | + |  |  |  | ✓ |
| **UL49-L** |  | 9554 | 10389-10394 | 10414 | + |  |  |  | ✓ |
| **UL50-L2** |  | 9651 | 8500-8505 | 8478 | - |  |  |  | ✓ |
| **UL27.5-S** |  | 17759 |  | 16869 | - |  | ✓ |  | ✓ |
| **UL27.5-L** |  | 18247 |  | 16869 | - |  |  |  | ✓ |
| **UL27-L** |  | 19964 |  | 16869 | - |  |  |  | ✓ |
| **UL33-L2-34-35** | 29701-29715 | 29743 | 32658-32663 | 32683 | + |  |  |  | ✓ |
| **UL31-L** |  | 29772 |  | 28694 | - |  |  |  | ✓ |
| **UL36.5-L1** |  | 35107 |  | 33127 | - |  |  |  | ✓ |
| **UL36.5-L2** |  | 35252 |  | 33127 | - |  |  |  | ✓ |
| **UL38-L2** |  | 44025 | 46486-46491 | 46527 | + |  | ✓ |  | ✓ |
| **UL38-L1** |  | 45217 | 46486-46491 | 46527 | + |  |  |  | ✓ |
| **UL39-40-L** | 46484-46498 | 46513 | 50117-50122 | 50136 | + |  |  |  | ✓ |
| **UL39.5-UL40-L2** |  | 47477 | 50117-50122 | 50136 | + |  |  |  | ✓ |
| **UL39.5-UL40-L1** |  | 47657 | 50117-50122 | 50136 | + |  |  |  | ✓ |
| **UL42-L1** | 51634-51648 | 51662 | 52954-52959 | 52960 | + |  |  |  | ✓ |
| **UL42-L2** |  | 51148 | 52937-52942 | 52958 | + |  |  | ✓ | ✓ |
| **UL41-L1** | 51731-51717 | 51696 |  | 50541 | - |  |  |  | ✓ |
| **UL42-43-AT** | 51721-51735 | 51749 |  | 54092 | + |  |  |  | ✓ |
| **UL42-43-L** |  | 50647 | 54102-54107 | 54119 | + |  |  | ✓ | ✓ |
| **UL43-44-AT** | 52955-52969 | 52970 | 55768-55773 | 55799 | + | ✓ |  |  | ✓ |
| **UL43-AT** | 52955-52969 | 52970 |  | 54065 | + |  |  |  | ✓ |
| **UL44-L2** |  | 54096 | 55660-55665 | 55685 | + |  |  |  | ✓ |
| **UL47-46-S** |  | 12098 | 16210-16215 | 16234 | + |  |  |  | ✓ |
| **CTO-L2** |  | 66327 |  | 63670 | - |  |  | ✓ |  |
| **UL21-L2** | 66754-66768 | 66789 |  | 64644 | - |  |  |  | ✓ |
| **UL10.4-L** |  | 81414 |  | 80906 | - |  | ✓ |  |  |
| **UL10-S** |  | 82105 |  | 80906 | - |  |  |  | ✓ |
| **UL10-L7** |  | 84325 |  | 80906 | - |  |  |  | ✓ |
| **UL7-L** |  | 88046 |  | 86675 | - |  |  |  | ✓ |
| **EP0.5-S1** |  | 97818 |  | 96421 | - |  |  |  | ✓ |
| **EP0-L1** |  | 98440 |  | 96421 | - |  |  |  | ✓ |
| **NOIR1-L6** |  | 107474 | 109278-109279 | 109304 | + |  |  |  | ✓ |
| **NOIR1-L4** |  | 107569 | 109278-109280 | 109304 | + |  |  |  | ✓ |
| **NOIR1-L5** |  | 107640 | 109278-109281 | 109304 | + |  |  |  | ✓ |
| **NOIR1-L3** |  | 107706 | 109278-109282 | 109304 | + |  |  |  | ✓ |
| **NOIR1-L2** |  | 107790 | 109278-109283 | 109304 | + |  |  |  | ✓ |
| **NOIR-2-AT** |  | 111580 |  | 110688 | - |  |  |  | ✓ |
| **US3-4-AT1** |  | 118336 |  | 121000 | + |  |  |  | ✓ |
| **AZURE-M** |  | 119305 |  | 117718 | - |  |  |  | ✓ |
| **US4-AT** | 119401-119415 | 119431 |  | 121915 | + |  |  |  | ✓ |
| **AZURE-L2** |  | 120177 |  | 117718 | - |  |  |  | ✓ |
| **US7-8-9-2-L** |  | 121756 |  | 126700 | + |  |  | ✓ |  |
| **US7-8-9-2-AT** |  | 122351 | 126995-127000 | 127019 | + | ✓ |  |  | ✓ |
| **US8-9-2-AT2** |  | 123547 | 126688-126693 | 126700 | + |  |  |  | ✓ |
| **US8-9-2-AT1** |  | 123547 | 126995-127000 | 127026 | + | ✓ |  |  | ✓ |
| **US2-AT2** | 125765-125779 | 125795 |  | 126816 | + |  |  |  | ✓ |
| **US1-L** |  | 130281 | 117375-117380 | 127330 | - |  |  |  | ✓ |
| **NCS4-AT1** | 119401-119415 | 119431 |  | 120523 | + |  |  |  | ✓ |
| **NCS4-AT2** | 119401-119415 | 119431 |  | 120489 | + |  |  |  | ✓ |
| **NOIR-1S2** |  | 135850 | 109278-109283 | 135433 | - |  | ✓ |  |  |
| **FORF15-L** |  | 74837 |  | 72049 | - |  |  |  | ✓ |

**Supplementary Table 4.** **Novel transcriptional overlaps between the novel PRV transcripts; and the previously detected and novel transcripts.**

| **Head-to-head** | | **Head-to-tail** | | **Tail-to-tail** | | |
| --- | --- | --- | --- | --- | --- | --- |
| **Transcripts** | **Overlap size** | **Transcripts** | **Overlap size** | | **Transcripts** | **Overlap size** |
| UL50L/UL49-L | 26 | UL27-AT1/UL27-L | 2902 | | UL51/UL50-L2 | 38 |
| UL50-L2/UL49-L | 97 | UL27-AT2/UL27-L | 2882 | | UL30/UL31.3 | 205 |
| UL32-S-31/UL32-35-C | 2046 | UL31.3/UL31.5 | 469 | | UL30/UL31.5 | 203 |
| UL32-S-31/UL33-L2-34-35 | 1396 | UL31.3/UL31-L | 469 | | UL30/UL31-L | 203 |
| UL31.3/UL32-35-C | 70 | UL38-L2/UL39-40-L | 14 | | UL35-AT/UL36.5-L1 | 23 |
| UL31/UL32-35-C | 581 | UL38-L1/UL39-40-L | 14 | | UL35-AT/UL36.5-L2 | 23 |
| UL32-31/UL32-35-C | 2212 | UL38/UL39-40-L | 14 | | UL43-44-AT/TRL26 | 5 |
| UL32-31/UL33-L2-34-35 | 1562 | NCL39/UL39.5-UL40-L2 | 1584 | | UL43-44-AT/UL26.3 | 5 |
| UL31.5/UL32-35-C | 272 | NCL39/UL39.5-UL40-L1 | 1404 | | UL9-8-L/UL7-L | 37 |
| UL31-L/UL32-35-C | 679 | UL41.5/NCL41 | 300 | | UL9-8/UL7-L | 37 |
| UL31-L/UL33-L2-34-35 | 29 | UL42-43-L/UL43-44-AT | 1149 | | UL8/UL7-L | 37 |
| UL37-36/UL38-L2 | 1357 | UL42-43-L/UL44-L2 | 23 | |  |  |
| UL37-36/UL38-L1 | 165 | UL42-L2/UL42-L1 | 1296 | |  |  |
| UL37/UL38-L2 | 1357 | UL42-L2/UL42-43-AT | 1209 | |  |  |
| UL37/UL38-L1 | 165 | UL42-L1/UL42-43-AT | 1211 | |  |  |
| UL41/UL42-L2 | 512 | UL42-43/UL43-44-AT | 1149 | |  |  |
| UL41/UL42-43-L | 1013 | UL42-43/UL44-L2 | 23 | |  |  |
| UL41.5/UL42-43-L | 363 | UL42-43-44/UL43-44-AT | 2715 | |  |  |
| UL41-L1/UL42-L1 | 34 | UL42-43-AT/UL43-44-AT | 1122 | |  |  |
| UL41-L1/UL42-L2 | 548 | UL43/UL44-L2 | 23 | |  |  |
| UL41-L1/UL42-43-L | 1049 | UL43S/UL44-L2 | 23 | |  |  |
| IE180/NOIR1-L6 | 483 | CTO-AT/CTO-L2 | 259 | |  |  |
| IE180/NOIR1-L4 | 388 | CTO-L/UL21-L2 | 1614 | |  |  |
| IE180/NOIR1-L5 | 317 | CTO-L/UL21-L3 | 1614 | |  |  |
| IE180/NOIR1-L3 | 251 | CTO-L2/UL21-L2 | 1683 | |  |  |
| IE180/NOIR1-L2 | 167 | CTO-L2/UL21-L3 | 1683 | |  |  |
| AZURE-S/US3-4-AT1 | 579 | UL21.5/NCL21 | 227 | |  |  |
| AZURE-L/NCS4-1 | 325 | UL19-18/UL18-17-16 | 972 | |  |  |
| AZURE-L/US3-4-AT1 | 1420 | UL18/UL18-17-16 | 972 | |  |  |
| AZURE-L/US4-AT | 325 | UL10.3/UL10.4 | 394 | |  |  |
| AZURE-L/NCS4-AT1 | 325 | UL10.3/UL10.4-L | 394 | |  |  |
| AZURE-L/NCS4-AT2 | 325 | UL10.3/UL10-S | 394 | |  |  |
| AZURE-M/US3-4-AT1 | 969 | UL10.3/UL10-L7 | 394 | |  |  |
| AZURE-L2/NCS4-1 | 746 | UL10.5/UL10-S | 715 | |  |  |
| AZURE-L2/US3-4-AT1 | 1841 | UL10.5/UL10-L7 | 715 | |  |  |
| AZURE-L2/US4-AT | 746 | UL10.6/UL10-S | 978 | |  |  |
| AZURE-L2/NCS4-AT1 | 746 | UL10.6/UL10-L7 | 978 | |  |  |
| AZURE-L2/NCS4-AT2 | 746 | UL10.7/UL10-S | 1100 | |  |  |
| US4-AS1/TRS4 | 229 | UL10.7/UL10-L7 | 1100 | |  |  |
| US4-AS1/US4-AT | 1480 | NCS1/TRS1 | 20 | |  |  |
|  |  | AZURE-L/US4-AS1 | 329 | |  |  |
|  |  | AZURE-L2/US4-AS1 | 750 | |  |  |
|  |  | US3-L-4/US4-AT | 1614 | |  |  |
|  |  | US3-4/US4-AT | 1614 | |  |  |
|  |  | US3-4-AT1/TRS4 | 318 | |  |  |
|  |  | US3-4-AT1/US4-AT | 1569 | |  |  |
|  |  | US4-6-7/US7-8-9-2-L | 1727 | |  |  |
|  |  | US4-6-7/US7-8-9-2-AT | 1132 | |  |  |
|  |  | US4-AT/US7-8-9-2-L | 159 | |  |  |
|  |  | US6/US7-8-9-2-L | 654 | |  |  |
|  |  | US6/US7-8-9-2-AT | 59 | |  |  |
|  |  | US6-7/US7-8-9-2-L | 1727 | |  |  |
|  |  | US6-7/US7-8-9-2-AT | 1132 | |  |  |
|  |  | US7-8-9-2-L/US7-8-9-2-AT | 4349 | |  |  |
|  |  | US7-8-9-2-L/US8-9-2-AT1 | 3153 | |  |  |
|  |  | US7-8-9-2-L/US2-AT2 | 905 | |  |  |
|  |  | US7-8-9/US8-9-2-AT2 | 2253 | |  |  |
|  |  | US7-8-9/US8-9-2-AT1 | 2253 | |  |  |
|  |  | US7-8-9/US2-AT2 | 5 | |  |  |
|  |  | US7-8-9-2/US8-9-2-AT1 | 3174 | |  |  |
|  |  | US7-8-9-2/US2-AT2 | 926 | |  |  |
|  |  | US7-8-9-2-AT/US8-9-2-AT1 | 3472 | |  |  |
|  |  | US8-L-9/US8-9-2-AT2 | 2253 | |  |  |
|  |  | US8-L-9/US8-9-2-AT1 | 2253 | |  |  |
|  |  | US8-L-9/US2-AT2 | 5 | |  |  |
|  |  | US8-9/US2-AT2 | 5 | |  |  |
|  |  | US8-9-2/US2-AT2 | 926 | |  |  |
|  |  | US8-9-2-AT2/US2-AT2 | 905 | |  |  |
|  |  | US9/US2-AT2 | 5 | |  |  |
|  |  | US9-2/US2-AT2 | 926 | |  |  |

**Supplementary Table 5. Putative uORFs of the PRV genome.**

| **AUG position** | **STOP position** | **Orientation** | **In transcript** | **Size** | **5' end distance** | **Kozak consensus score** |
| --- | --- | --- | --- | --- | --- | --- |
| 339 | 680 | + | ORF-1L2 | 342 | 178 |  |
| 339 | 680 | + | ORF-1L1 | 342 | 16 |  |
| 377 | 1054 | + | ORF-1L2 | 678 | 216 |  |
| 377 | 1054 | + | ORF-1L1 | 678 | 54 |  |
| 383 | 1054 | + | ORF-1L2 | 672 | 222 |  |
| 383 | 1054 | + | ORF-1L1 | 672 | 60 |  |
| 492 | 680 | + | ORF-1L2 | 189 | 331 |  |
| 492 | 680 | + | ORF-1L1 | 189 | 169 |  |
| 1051 | 1233 | + | ORF-1L2 | 183 | 890 |  |
| 1051 | 1233 | + | ORF-1L1 | 183 | 728 |  |
| 1051 | 1233 | + | ORF-1M2 | 183 | 62 |  |
| 1090 | 1233 | + | ORF-1L2 | 144 | 929 |  |
| 1090 | 1233 | + | ORF-1L1 | 144 | 767 |  |
| 1090 | 1233 | + | ORF-1M2 | 144 | 101 |  |
| 1129 | 1233 | + | ORF-1L2 | 105 | 968 |  |
| 1129 | 1233 | + | ORF-1L1 | 105 | 806 |  |
| 1129 | 1233 | + | ORF-1M2 | 105 | 140 |  |
| 1129 | 1233 | + | orf1-M-2-S | 105 | 25 |  |
| 1233 | 1982 | + | ORF-1L2 | 750 | 1072 |  |
| 1233 | 1982 | + | ORF-1L1 | 750 | 910 |  |
| 1233 | 1982 | + | ORF-1M2 | 750 | 244 |  |
| 1233 | 1982 | + | orf1-M-2-S | 750 | 129 |  |
| 1269 | 1982 | + | ORF-1L2 | 714 | 1108 |  |
| 1269 | 1982 | + | ORF-1L1 | 714 | 946 |  |
| 1269 | 1982 | + | ORF-1M2 | 714 | 280 |  |
| 1269 | 1982 | + | orf1-M-2-S | 714 | 165 |  |
| 1419 | 1982 | + | ORF-1L2 | 564 | 1258 |  |
| 1419 | 1982 | + | ORF-1L1 | 564 | 1096 |  |
| 1419 | 1982 | + | ORF-1M2 | 564 | 430 |  |
| 1419 | 1982 | + | orf1-M-2-S | 564 | 315 |  |
| 1419 | 1982 | + | ORF-1M1 | 564 | 83 |  |
| 9509 | 9438 | - | ul50-L-2 | 72 | 142 |  |
| 9509 | 9438 | - | ul50L | 72 | 71 |  |
| 9575 | 9610 | + | ul49-L | 36 | 21 |  |
| 9649 | 9230 | - | ul50-L-2 | 420 | 2 |  |
| 19891 | 19544 | - | ul27-L | 348 | 73 |  |
| 25751 | 25719 | - | ul29-L | 33 | 396 |  |
| 25850 | 25824 | - | ul29-L | 27 | 297 |  |
| 25952 | 25824 | - | ul29-L | 129 | 195 |  |
| 44761 | 44769 | + | ul38-L-2 | 9 | 736 |  |
| 44914 | 45114 | + | ul38-L-2 | 201 | 889 |  |
| 46628 | 49135 | + | ul39-40-L | 2508 | 115 |  |
| 46763 | 49135 | + | ul39-40-L | 2373 | 250 | 47 |
| 47225 | 49135 | + | ul39-40-L | 1911 | 712 |  |
| 47264 | 49135 | + | ul39-40-L | 1872 | 751 |  |
| 47321 | 49135 | + | ul39-40-L | 1815 | 808 |  |
| 47420 | 49135 | + | ul39-40-L | 1716 | 907 |  |
| 47612 | 49135 | + | ul39-40-L | 1524 | 1099 |  |
| 47612 | 49135 | + | ul39.5-ul40-L-2 | 1524 | 135 |  |
| 47639 | 49135 | + | ul39-40-L | 1497 | 1126 |  |
| 47639 | 49135 | + | ul39.5-ul40-L-2 | 1497 | 162 |  |
| 47732 | 49135 | + | ul39-40-L | 1404 | 1219 |  |
| 47732 | 49135 | + | ul39.5-ul40-L-2 | 1404 | 255 |  |
| 47732 | 49135 | + | ul39.5-ul40-L-1 | 1404 | 75 |  |
| 47741 | 49135 | + | ul39-40-L | 1395 | 1228 |  |
| 47741 | 49135 | + | ul39.5-ul40-L-2 | 1395 | 264 |  |
| 47741 | 49135 | + | ul39.5-ul40-L-1 | 1395 | 84 |  |
| 47975 | 49135 | + | ul39-40-L | 1161 | 1462 |  |
| 47975 | 49135 | + | ul39.5-ul40-L-2 | 1161 | 498 |  |
| 47975 | 49135 | + | ul39.5-ul40-L-1 | 1161 | 318 |  |
| 48005 | 49135 | + | ul39-40-L | 1131 | 1492 |  |
| 48005 | 49135 | + | ul39.5-ul40-L-2 | 1131 | 528 |  |
| 48005 | 49135 | + | ul39.5-ul40-L-1 | 1131 | 348 |  |
| 48026 | 49135 | + | ul39-40-L | 1110 | 1513 |  |
| 48026 | 49135 | + | ul39.5-ul40-L-2 | 1110 | 549 |  |
| 48026 | 49135 | + | ul39.5-ul40-L-1 | 1110 | 369 |  |
| 48029 | 49135 | + | ul39-40-L | 1107 | 1516 |  |
| 48029 | 49135 | + | ul39.5-ul40-L-2 | 1107 | 552 |  |
| 48029 | 49135 | + | ul39.5-ul40-L-1 | 1107 | 372 |  |
| 48248 | 49135 | + | ul39-40-L | 888 | 1735 |  |
| 48248 | 49135 | + | ul39.5-ul40-L-2 | 888 | 771 |  |
| 48248 | 49135 | + | ul39.5-ul40-L-1 | 888 | 591 |  |
| 48251 | 49135 | + | ul39-40-L | 885 | 1738 |  |
| 48251 | 49135 | + | ul39.5-ul40-L-2 | 885 | 774 |  |
| 48251 | 49135 | + | ul39.5-ul40-L-1 | 885 | 594 |  |
| 48359 | 49135 | + | ul39-40-L | 777 | 1846 |  |
| 48359 | 49135 | + | ul39.5-ul40-L-2 | 777 | 882 |  |
| 48359 | 49135 | + | ul39.5-ul40-L-1 | 777 | 702 |  |
| 48365 | 49135 | + | ul39-40-L | 771 | 1852 |  |
| 48365 | 49135 | + | ul39.5-ul40-L-2 | 771 | 888 |  |
| 48365 | 49135 | + | ul39.5-ul40-L-1 | 771 | 708 |  |
| 48428 | 49135 | + | ul39-40-L | 708 | 1915 |  |
| 48428 | 49135 | + | ul39.5-ul40-L-2 | 708 | 951 |  |
| 48428 | 49135 | + | ul39.5-ul40-L-1 | 708 | 771 |  |
| 48509 | 49135 | + | ul39-40-L | 627 | 1996 |  |
| 48509 | 49135 | + | ul39.5-ul40-L-2 | 627 | 1032 |  |
| 48509 | 49135 | + | ul39.5-ul40-L-1 | 627 | 852 |  |
| 48551 | 49135 | + | ul39-40-L | 585 | 2038 |  |
| 48551 | 49135 | + | ul39.5-ul40-L-2 | 585 | 1074 |  |
| 48551 | 49135 | + | ul39.5-ul40-L-1 | 585 | 894 |  |
| 48620 | 49135 | + | ul39-40-L | 516 | 2107 |  |
| 48620 | 49135 | + | ul39.5-ul40-L-2 | 516 | 1143 |  |
| 48620 | 49135 | + | ul39.5-ul40-L-1 | 516 | 963 |  |
| 48686 | 49135 | + | ul39-40-L | 450 | 2173 |  |
| 48686 | 49135 | + | ul39.5-ul40-L-2 | 450 | 1209 |  |
| 48686 | 49135 | + | ul39.5-ul40-L-1 | 450 | 1029 |  |
| 48740 | 49135 | + | ul39-40-L | 396 | 2227 |  |
| 48740 | 49135 | + | ul39.5-ul40-L-2 | 396 | 1263 |  |
| 48740 | 49135 | + | ul39.5-ul40-L-1 | 396 | 1083 |  |
| 48911 | 49135 | + | ul39-40-L | 225 | 2398 |  |
| 48911 | 49135 | + | ul39.5-ul40-L-2 | 225 | 1434 |  |
| 48911 | 49135 | + | ul39.5-ul40-L-1 | 225 | 1254 |  |
| 48953 | 49135 | + | ul39-40-L | 183 | 2440 |  |
| 48953 | 49135 | + | ul39.5-ul40-L-2 | 183 | 1476 |  |
| 48953 | 49135 | + | ul39.5-ul40-L-1 | 183 | 1296 |  |
| 49007 | 49135 | + | ul39-40-L | 129 | 2494 |  |
| 49007 | 49135 | + | ul39.5-ul40-L-2 | 129 | 1530 |  |
| 49007 | 49135 | + | ul39.5-ul40-L-1 | 129 | 1350 |  |
| 49019 | 49135 | + | ul39-40-L | 117 | 2506 |  |
| 49019 | 49135 | + | ul39.5-ul40-L-2 | 117 | 1542 |  |
| 49019 | 49135 | + | ul39.5-ul40-L-1 | 117 | 1362 |  |
| 49052 | 49135 | + | ul39-40-L | 84 | 2539 |  |
| 49052 | 49135 | + | ul39.5-ul40-L-2 | 84 | 1575 |  |
| 49052 | 49135 | + | ul39.5-ul40-L-1 | 84 | 1395 |  |
| 51695 | 51856 | + | ul42-L-1 | 162 | 33 |  |
| 54155 | 54637 | + | ul44-L2 | 483 | 59 |  |
| 54155 | 54637 | + | ul44-L | 483 | 24 |  |
| 59010 | 59318 | + | ul23-L3 | 309 | 28 |  |
| 59070 | 59318 | + | ul23-L3 | 249 | 88 |  |
| 59085 | 59318 | + | ul23-L3 | 234 | 103 |  |
| 59276 | 59629 | + | ul23-L3 | 354 | 294 |  |
| 59276 | 59629 | + | ul23-L2 | 354 | 135 |  |
| 59453 | 59629 | + | ul23-L3 | 177 | 471 |  |
| 59453 | 59629 | + | ul23-L2 | 177 | 312 |  |
| 66808 | 66716 | - | ul21-L-3 | 93 | 376 |  |
| 66811 | 66716 | - | ul21-L-3 | 96 | 373 |  |
| 66815 | 66216 | - | ul21-L-3 | 600 | 369 |  |
| 66921 | 66808 | - | ul21-L-3 | 114 | 263 |  |
| 66951 | 66808 | - | ul21-L-3 | 144 | 233 |  |
| 66984 | 66808 | - | ul21-L-3 | 177 | 200 |  |
| 67155 | 67135 | - | ul21-L-3 | 21 | 29 |  |
| 82104 | 84635 | + | ul9-8-L | 2532 | 175 |  |
| 82185 | 84635 | + | ul9-8-L | 2451 | 256 |  |
| 82326 | 84635 | + | ul9-8-L | 2310 | 397 |  |
| 82358 | 82387 | + | ul9-8-L | 30 | 429 |  |
| 82389 | 84635 | + | ul9-8-L | 2247 | 460 |  |
| 82446 | 84635 | + | ul9-8-L | 2190 | 517 |  |
| 82494 | 82423 | - | ul10-L-7 | 72 | 1831 |  |
| 82494 | 82423 | - | ul10-L4 | 72 | 84 |  |
| 82494 | 82423 | - | ul10-L5 | 72 | 153 |  |
| 82500 | 84635 | + | ul9-8-L | 2136 | 571 |  |
| 82605 | 84635 | + | ul9-8-L | 2031 | 676 |  |
| 82606 | 82577 | - | ul10-L-7 | 30 | 1719 |  |
| 82606 | 82577 | - | ul10-L5 | 30 | 41 |  |
| 82638 | 84635 | + | ul9-8-L | 1998 | 709 | 42 |
| 82639 | 82625 | - | ul10-L-7 | 15 | 1686 |  |
| 82639 | 82625 | - | ul10-L5 | 15 | 8 |  |
| 82707 | 84635 | + | ul9-8-L | 1929 | 778 |  |
| 82708 | 82625 | - | ul10-L-7 | 84 | 1617 |  |
| 83223 | 84635 | + | ul9-8-L | 1413 | 1294 |  |
| 83224 | 83216 | - | ul10-L-7 | 9 | 1101 |  |
| 83244 | 84635 | + | ul9-8-L | 1392 | 1315 |  |
| 83262 | 84635 | + | ul9-8-L | 1374 | 1333 |  |
| 83263 | 83231 | - | ul10-L-7 | 33 | 1062 |  |
| 83359 | 83273 | - | ul10-L-7 | 87 | 966 |  |
| 83487 | 84635 | + | ul9-8-L | 1149 | 1558 |  |
| 83566 | 83273 | - | ul10-L-7 | 294 | 759 |  |
| 83740 | 83273 | - | ul10-L-7 | 468 | 585 |  |
| 84294 | 84635 | + | ul9-8-L | 342 | 2365 |  |
| 84459 | 84635 | + | ul9-8-L | 177 | 2530 |  |
| 84525 | 84635 | + | ul9-8-L | 111 | 2596 |  |
| 88028 | 87540 | - | ul7-L | 489 | 18 |  |
| 89470 | 91974 | + | ul5-4-L | 2505 | 95 | 38 |
| 89536 | 91974 | + | ul5-4-L | 2439 | 161 |  |
| 89699 | 89833 | + | ul5-4-L | 135 | 324 |  |
| 89699 | 89833 | + | ul5-4-M | 135 | 19 |  |
| 89746 | 91974 | + | ul5-4-L | 2229 | 371 |  |
| 89746 | 91974 | + | ul5-4-M | 2229 | 66 |  |
| 90445 | 91974 | + | ul5-4-L | 1530 | 1070 |  |
| 90445 | 91974 | + | ul5-4-M | 1530 | 765 |  |
| 90595 | 91974 | + | ul5-4-L | 1380 | 1220 |  |
| 90595 | 91974 | + | ul5-4-M | 1380 | 915 |  |
| 91036 | 91974 | + | ul5-4-L | 939 | 1661 |  |
| 91036 | 91974 | + | ul5-4-M | 939 | 1356 |  |
| 91252 | 91974 | + | ul5-4-L | 723 | 1877 |  |
| 91252 | 91974 | + | ul5-4-M | 723 | 1572 |  |
| 91261 | 91974 | + | ul5-4-L | 714 | 1886 |  |
| 91261 | 91974 | + | ul5-4-M | 714 | 1581 |  |
| 91327 | 91974 | + | ul5-4-L | 648 | 1952 |  |
| 91327 | 91974 | + | ul5-4-M | 648 | 1647 |  |
| 91450 | 91974 | + | ul5-4-L | 525 | 2075 |  |
| 91450 | 91974 | + | ul5-4-M | 525 | 1770 |  |
| 91705 | 91974 | + | ul5-4-L | 270 | 2330 |  |
| 91705 | 91974 | + | ul5-4-M | 270 | 2025 |  |
| 91747 | 91974 | + | ul5-4-L | 228 | 2372 |  |
| 91747 | 91974 | + | ul5-4-M | 228 | 2067 |  |
| 91819 | 91974 | + | ul5-4-L | 156 | 2444 |  |
| 91819 | 91974 | + | ul5-4-M | 156 | 2139 |  |
| 91840 | 91974 | + | ul5-4-L | 135 | 2465 |  |
| 91840 | 91974 | + | ul5-4-M | 135 | 2160 |  |
| 91873 | 91974 | + | ul5-4-L | 102 | 2498 |  |
| 91873 | 91974 | + | ul5-4-M | 102 | 2193 |  |
| 91879 | 91974 | + | ul5-4-L | 96 | 2504 |  |
| 91879 | 91974 | + | ul5-4-M | 96 | 2199 |  |
| 98189 | 97905 | - | ep0-L-1 | 285 | 251 |  |
| 98192 | 97905 | - | ep0-L-1 | 288 | 248 |  |
| 98210 | 97905 | - | ep0-L-1 | 306 | 230 |  |
| 98421 | 98299 | - | ep0-L-1 | 123 | 19 |  |
| 98433 | 98299 | - | ep0-L-1 | 135 | 7 |  |
| 118238 | 119404 | + | US3-L-4 | 1167 | 62 |  |
| 118247 | 119404 | + | US3-L-4 | 1158 | 71 |  |
| 118255 | 118311 | + | US3-L-4 | 57 | 79 |  |
| 118400 | 119404 | + | US3-L-4 | 1005 | 224 |  |
| 118503 | 118517 | + | US3-L-4 | 15 | 327 |  |
| 118670 | 119404 | + | US3-L-4 | 735 | 494 |  |
| 118679 | 119404 | + | US3-L-4 | 726 | 503 |  |
| 118712 | 119404 | + | US3-L-4 | 693 | 536 |  |
| 118718 | 119404 | + | US3-L-4 | 687 | 542 |  |
| 118727 | 119404 | + | US3-L-4 | 678 | 551 |  |
| 118799 | 119404 | + | US3-L-4 | 606 | 623 |  |
| 118820 | 119404 | + | US3-L-4 | 585 | 644 |  |
| 118880 | 119404 | + | US3-L-4 | 525 | 704 |  |
| 118889 | 119404 | + | US3-L-4 | 516 | 713 |  |
| 119156 | 119404 | + | US3-L-4 | 249 | 980 |  |
| 119333 | 119404 | + | US3-L-4 | 72 | 1157 |  |
| 119370 | 119744 | + | US3-L-4 | 375 | 1194 |  |
| 119390 | 119404 | + | US3-L-4 | 15 | 1214 |  |
| 123568 | 125301 | + | US8-L-9 | 1734 | 77 |  |
| 123731 | 123799 | + | US8-L-9 | 69 | 240 |  |
| 124300 | 125301 | + | US8-L-9 | 1002 | 809 |  |
| 124321 | 125301 | + | US8-L-9 | 981 | 830 |  |
| 124633 | 125301 | + | US8-L-9 | 669 | 1142 |  |
| 124882 | 125301 | + | US8-L-9 | 420 | 1391 |  |
| 124984 | 125301 | + | US8-L-9 | 318 | 1493 |  |
| 125246 | 125266 | + | US8-L-9 | 21 | 1755 |  |
| 125258 | 125266 | + | US8-L-9 | 9 | 1767 |  |
| 125285 | 125572 | + | US8-L-9 | 288 | 1794 |  |
| 129138 | 128968 | - | us1-L | 171 | 1143 |  |
| 129363 | 128968 | - | us1-L | 396 | 918 |  |
| 129414 | 128968 | - | us1-L | 447 | 867 |  |
| 129693 | 129556 | - | us1-L | 138 | 588 |  |
| 129974 | 129825 | - | us1-L | 150 | 307 |  |
| 130235 | 130128 | - | us1-L | 108 | 46 |  |
