# Supplementary figures and images for: Multi-Platform Sequencing Approach Reveals a Novel Transcriptome Profile in Pseudorabies Virus
Source: Front Microbiol. 2018 Jan 22;8:2708. doi: 10.3389/fmicb.2017.02708 (PMC5786565; doi:10.3389/fmicb.2017.02708)

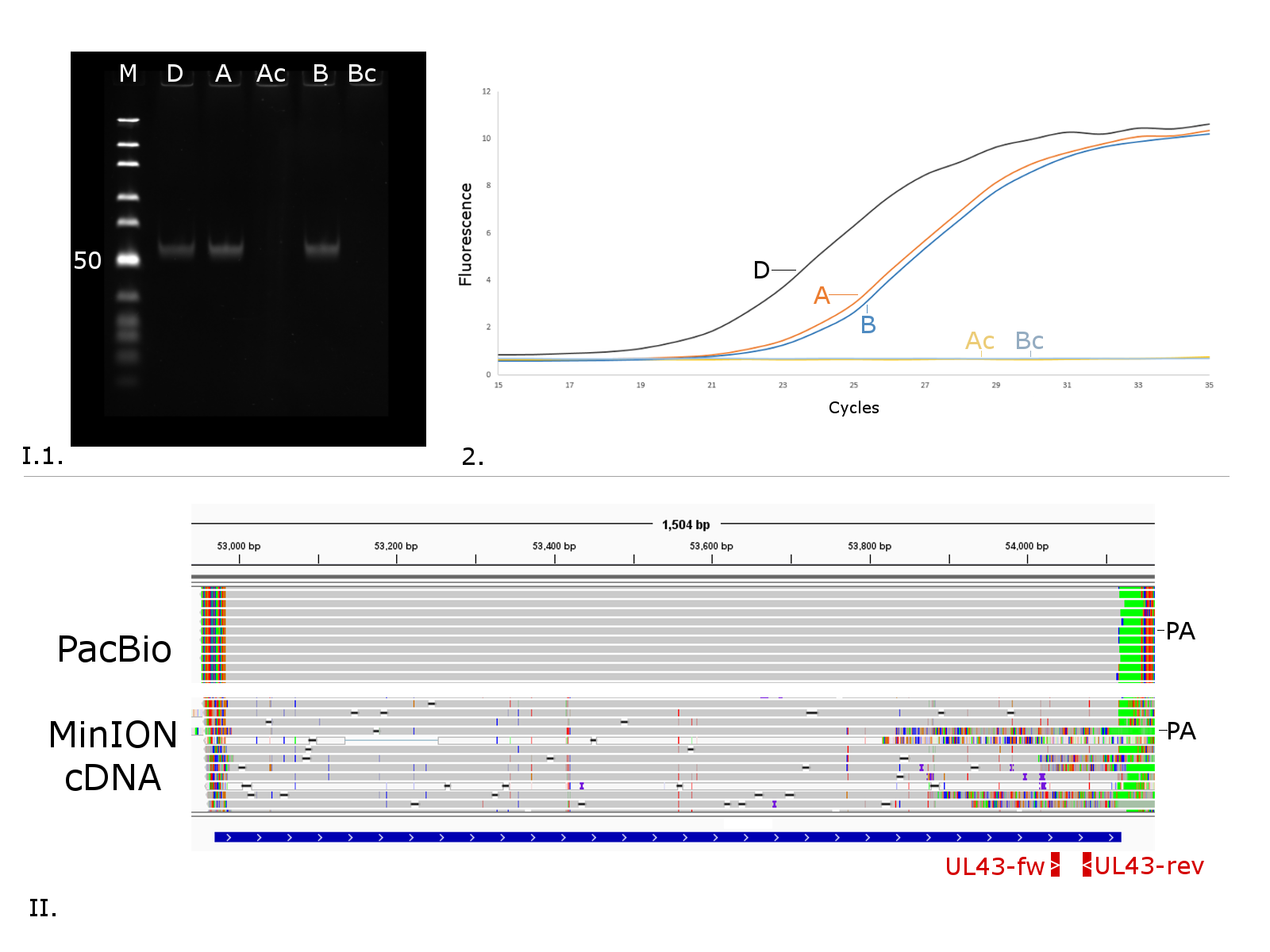

Supplement: Supplementary file 2 [file Image_1.TIF]
